# Supplementary material for: Prognostic Value of N-Terminal Pro-Brain Natriuretic Peptide and High-Sensitivity Troponin T Levels in the Natural History of Transthyretin Amyloid Cardiomyopathy and Their Evolution after Tafamidis Treatment
Source: J Clin Med. 2021 Oct 22;10(21):4868. doi: 10.3390/jcm10214868 (PMC8584290; doi:10.3390/jcm10214868)
Supplement: Supplementary file 1 [file jcm-10-04868-s001.zip › jcm-1407262-supplementary.pdf]

Table S1 Supp shows time intervals of NT-proBNP and cTnT-HS assessments before and after initiating tafamidis in Cohort B.

Table S1. Time intervals of NT-proBNP and cTnT-HS assessments before and after initiating tafamidis in Cohort B (n=248)

	**Tafamidis +/- 30 days**	**At 6 months (±3)**	**At 12 months (±3)**	**At 18 months (±3)**
**Months From Tafamidis Initiation to NT-proBNP assessment for non-dead patients, median (IQR)**	0	5.6 (5.5 - 5.8)	12 (11.8 - 12.1)	17,8 (17.5 - 18.1)
**Months From Tafamidis Initiation to cTnT-HS assessment for non-dead patients, median (IQR)**	0	5.6 (5.4 – 5.8)	12.0 (11.8 – 12.1)	17.8 (15.4 – 18.1)
